# lncRNA-ES3/miR-34c-5p/BMF axis is involved in regulating high-glucose-induced calcification/senescence of VSMCs

**DOI:** 10.18632/aging.101758

**Published:** 2019-01-17

**Authors:** Xiao Lin, Jun-Kun Zhan, Jia-Yu Zhong, Yan-Jiao Wang, Yi Wang, Shuang Li, Jie-Yu He, Pan Tan, Yi-Yin Chen, Xue-Bin Liu, Xing-Jun Cui, You-Shuo Liu

**Affiliations:** ^1^ Department of Geriatrics, Institute of Aging and Geriatrics, The Second Xiangya Hospital, Central South University, Changsha, Hunan 410011, China; ^*^ Equal contribution

**Keywords:** miR-34c-5p, lncRNA-ES3, BMF, VSMCs calcification/senescence, vascular aging, diabetes

## Abstract

Vascular calcification/aging is common in diabetes and is associated with increased morbidity and mortality of patients. MiR-34c-5p, not miR-34c-3p, was suppressed significantly in calcification/senescence of human aorta vascular smooth muscle cells (HA-VSMCs) induced by high glucose, which was proven by the formation of mineralized nodules and staining of senescence associated-β-galactosidase staining (SA β-gal) positive cells. Overexpression of miR-34c-5p alleviated calcification/senescence of HA-VSMCs, whereas inhibition of miR-34c-5p received the opposite results. Bcl-2 modifying factor (BMF) was a functional target of miR-34c-5p and it was involved in the process of calcification/senescence of HA-VSMCs. Besides, lncRNA-ES3 acted as a competing endogenous RNAs (ceRNA) of miR-34c-5p to enhance BMF expression. Further, lncRNA-ES3 inhibited miR-34c-5p expression by direct interaction and its knockdown suppressed the calcification/senescence of HA-VSMCs. Our results showed for the first time that the calcification/senescence of VSMCs was regulated by lncRNA-ES3 /miR-34c-5p/BMF axis.

## INTRODUCTION

Vascular aging is very common in patients with diabetes and it can influence the threshold, progression, severity, and prognosis of the cardiovascular disease [1]. Vascular calcification is an important phenotype of vascular aging and is one of the common effects of macrovascular complications in patients with diabetes, mainly involving the media of artery, also termed Monckeberg’s arterial calcification [2]. Furthermore, diabetic artery calcification/aging leads to arteriosclerosis, amputations, kidney failure, stroke, and increased incidence of cardiovascular events and mortality [3, 4]. Hyperglycemia is the main characteristic of diabetes and increasing evidence has demonstrated that high glucose is an important regulator of endothelial cell senescence, wherein accumulative premature senescent cells participate in the onset and progress of diabetic vascular aging [5, 6]. However, very few reports have studied the effect of high glucose on calcification/senescence of vascular smooth muscle cells (VSMCs). Moreover, the mechanisms involved in high glucose-induced VSMC calcification/senescence remain unclear.

MicroRNAs (miRNAs) are a class of small noncoding RNAs with the length of ~22 nt. Presently, miRNAs have been verified to control gene expression by binding to 3’ untranslated regions (UTR) of target genes, and the impacts of miRNAs on regulating the proliferation, migration, differentiation, calcification, and apoptosis of VSMCs have been investigated in numerous fields [7, 8]. MiR-34c, a member of miR-34 family, is reported to participate in osteoblast differentiation [9], VSMC calcification [10], endothelial senescence [11], and many others. However, the detailed mechanism of the function of miR-34c in VSMC calcification/ senescence is not yet fully understood.

It is well known that miRNAs exerted their functions by regulating translation or stability of target mRNAs [7, 9, 12]. Bcl-2 modifying factor (BMF) has been reported to play an important role in the regulation of cell functions under hyperglycemia [13–15]. For example, Liu et al. found that miR-34c could increase the expression of Bcl-2 and inhibit the apoptosis of renal podocytes in a high glucose environment [13]. Garnet et al. confirmed that BMF could promote apoptosis of proximal renal tubular cells in mice with diabetic nephropathy [14]. In addition, BMF could also promote VSMC apoptosis by regulating miR-221/222 expression [15]. However, the role of BMF in regulating VSMC calcification/senescence still needs to be further explored.

Long noncoding RNAs (lncRNAs), a class of noncoding RNAs longer than 200 nt in length, are actively regulated during cellular senescence [16–18]. They can regulate gene expression post-transcriptionally by base-pairing with mRNAs to modulate their translation and/or stability [19, 20]. Competing endogenous RNAs (ceRNAs) are stable lncRNAs that accumulate in large numbers and modulate gene expression in different ways, including decoys or sponges for miRNAs [21, 22]. For instance, Lv et al. demonstrated that lncRNA H19/miR-675/PTEN was the signaling axis in SMC proliferation [19]. Another study showed that H19 facilitated proliferation and inhibited apoptosis by sponging to miR-148b in ox-LDL-stimulated HA-VSMCs [20]. However, there are still plenty of lncRNAs whose functions are not yet reported and need to be further studied, including lncRNA-ES3 (LINC00458). It is not clear whether lncRNA-ES3 plays a role in cellular senescence with diabetes, and that the relationship between lncRNA-ES3 and miR-34c in VSMC senescence needs to be demonstrated.

In the present study, we found that BMF was the target gene of miR-34c-5p, and that lncRNA-ES3 also had some complementary sites for miR-34c-5p. Thus, we aimed to further explore the underlying roles and molecular mechanisms of lncRNA-ES3/miR-34c-5p/BMF in VSMCs calcification/senescence and expected to discover potential therapeutic targets for diabetic vascular aging.

## RESULTS

### The expression of miR-34c-5p was decreased in high glucose-induced HA-VSMCs calcification/senescence

**Figure 1:**
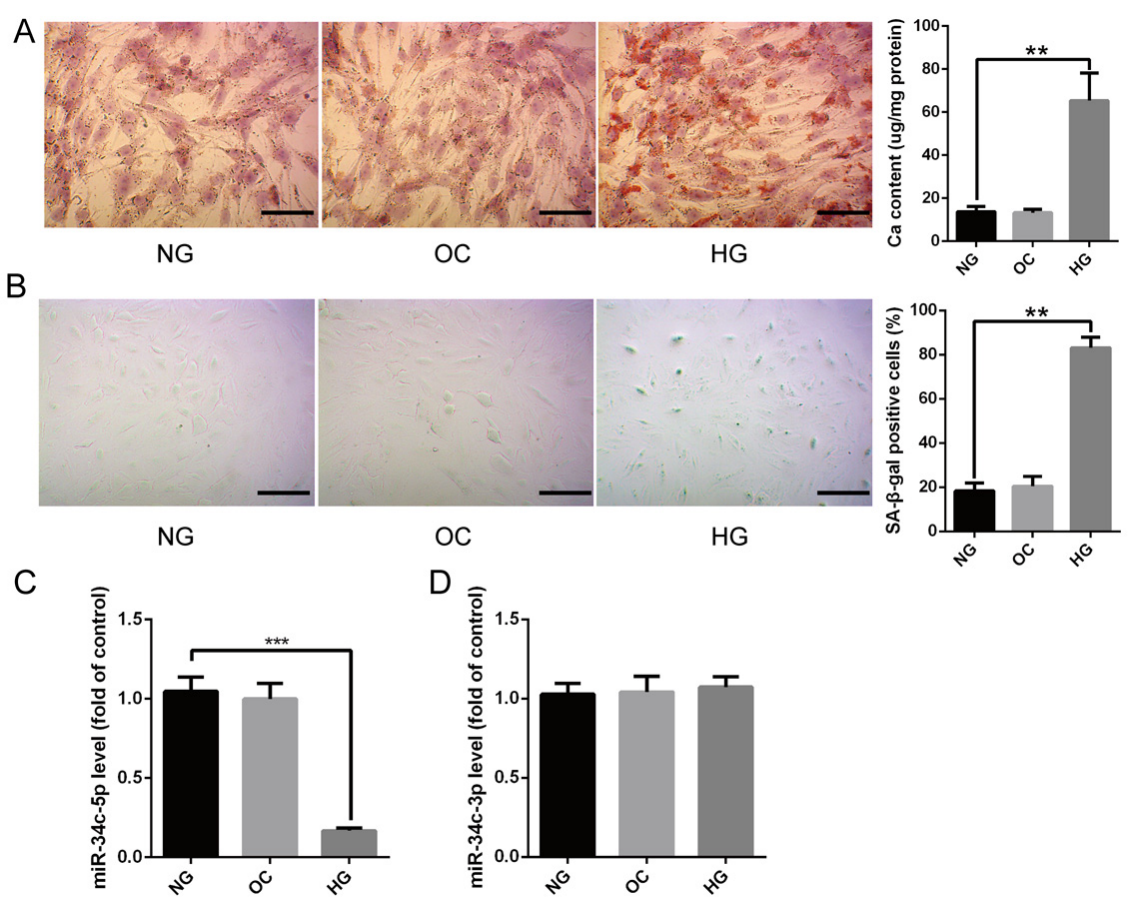
**The expression of miR-34c-5p in HG-induced HA-VSMCs.** (**A**) HA-VSMCs were treated with NG, OC, or HG for 14 days and then subjected to Alizarin Red S staining. The calcium content was extracted with cetylpyridinium chloride and quantified by spectrophotometry. Representative pictures are shown and the scale bar is 100 μm. (**B**) HA-VSMCs were treated with NG, OC, or HG for 72 hours and then subjected to SA-β-gal staining. Semi-quantitative analysis of SA-β-gal positive cells were performed using Image J. (**C** and **D**) qRT-PCR showing the expression of miR-3c-5p and miR-34c-3p in the above three groups. The data are expressed as mean ± SD, n=3, **p<0.005, ***p<0.0005. NG: normal glucose; OC: osmolarity control; HG: high glucose.

Mineralized nodule formations revealed by Alizarin Red S staining were increased greatly in high glucose (HG)-induced HA-VSMCs for 14 days (Figure 1A), and SA-β-gal staining showed that the SA-β-gal positive cells were significantly increased as well (Figure 1B). Osmolarity control (OC), however, had no significant effects on calcification/senescence of HA-VSMCs (Figure 1A and 1B). qRT-PCR revealed that the level of miR-34c-5p was strikingly decreased in HA-VSMCs treated with HG (Figure 1C). Nevertheless, the level of miR-34c-3p had no significant difference between HA-VSMCs treated with normal glucose (NG) and those with HG (Figure 1D). Moreover, OC had no significant effect on expression of both miR-34c-5p and miR-34c-3p compared with NG. These results suggested that miR-34c-5p might be involved in regulating HA-VSMCs calcification/senescence.

### miR-34c-5p was involved in attenuating calcification/senescence of HA-VSMCs

**Figure 2:**
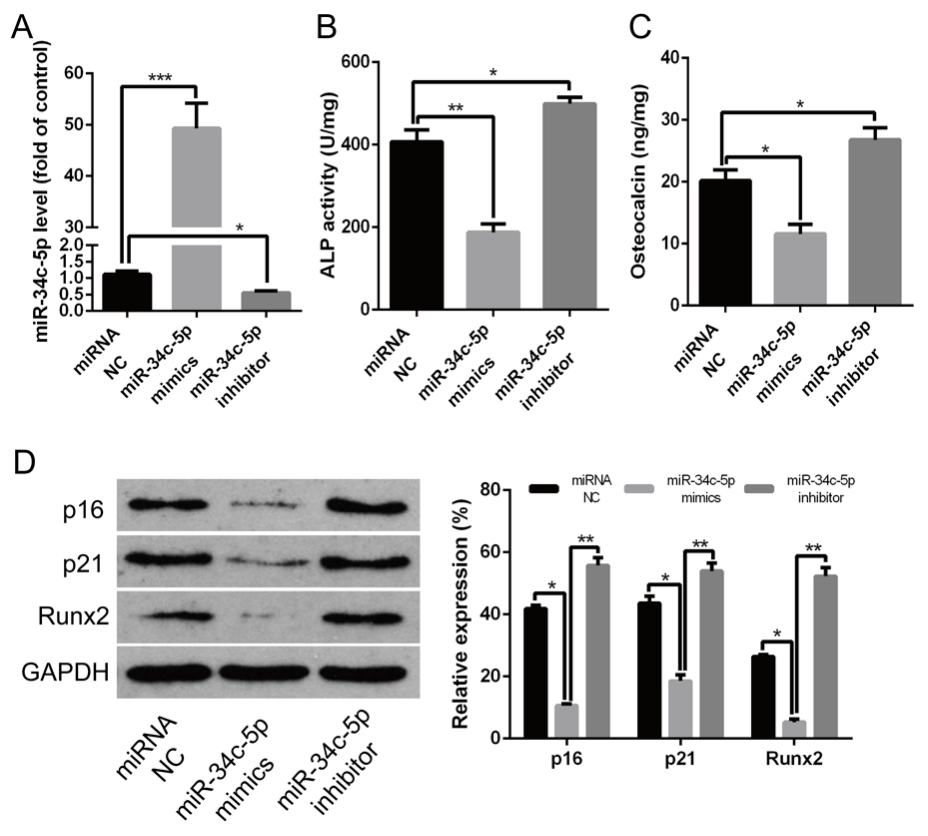
**miR-34c-5p inhibiting the calcification/senescence of HA-VSMCs.** (**A**) HA-VSMCs was transfected with miRNA NC, miR-34c-5p mimics, and miR-34c-5p inhibitor, and subjected to qRT–PCR analysis of miR-34c-5p. (**B–D**) HA-VSMCs were transfected with miRNA NC, miR-34c-5p mimics, and miR-34c-5p inhibitor, respectively. Then, ALP activity, OC secretion, and Runx2, p16, and p21 protein levels were measured. The data are expressed as mean ± SD, n=3, *p<0.05, **p<0.005, ***p<0.0005. NC: negative control.

In order to demonstrate whether miR-34c-5p was involved in regulating calcification/senescence of HA-VSMCs, the gain- and loss-of-function approach were used to overexpress or inhibit the expression of miR-34c-5p, respectively. MiR-34c-5p mimics and inhibitor were transfected into HA-VSMCs to overexpress or inhibit expression of miR-34c-5p. The qRT-PCR results showed that the miR-34c-5p mimics induced miR-34c-5p levels by about 50-fold and the miR-34c-5p inhibitor decreased the expression of miR-34c-5p significantly (Figure 2A). Meanwhile, the ALP activity, OC secretion, and Runx2 protein levels were significantly decreased when overexpressing miR-34c-5p, whereas the opposite occurred when inhibiting its expression (Figure 2B–2D). In addition, the overexpression of miR-34c-5p significantly decreased the level of senescence markers p16 and p21 proteins, whereas inhibiting the expression of miR-34c-5p produced the opposite results (Figure 2D). Therefore, these data indicated that miR-34c-5p plays a negative role in the process of calcification/senescence of HA-VSMCs.

### lncRNA-ES3 suppressed miR-34c-5p expression by direct interaction

**Figure 3:**
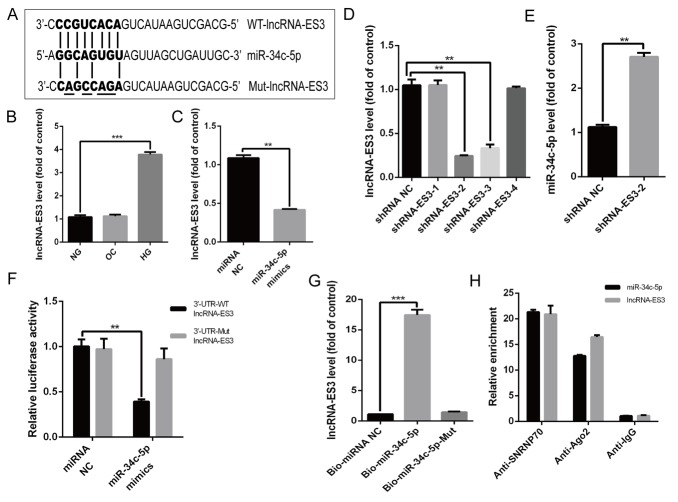
**lncRNA-ES3 suppressed miR-34c-5p expression by direct interaction.** (**A**) Schematic representation of the putative binding sites between lncRNA-ES3 and miR-34c-5p, and the mutant sites in Mut-lncRNA-ES3 reporter were underlined. (**B**) qRT-PCR showed the expression of lncRNA-ES3 in HA-VSMCs cultured with NG, OC, and HG. (**C**) HA-VSMCs was transfected with miRNA NC and miR-34c-5p mimics and harvested for the examination of lncRNA-ES3 by qRT-PCR. (**D**) The inhibitory efficiency of shRNAs targeting lncRNA-ES3 was verified by qRT-PCR. (**E**) HA-VSMCs were transfected with shRNA NC and shRNA-ES3-2, and the expression of miR-34c-5p was detected by qRT-PCR. (**F**) The WT-lncRNA-ES3 3’UTR and the Mut-lncRNA-ES3 3’UTR reporters were co-transfected with miR-34c-5p mimics or control oligos into HA-VSMCs. Forty-eight hours after transfection, luciferase activities were measured. (**G**) The expression of lncRNA-ES3 was detected by qRT-PCR after biotin-labeled miR-34c-5p pull-down assay. (**H**) RIP and qRT-PCR assays were performed to explore the binding efficiency of miR-34c-5p and lncRNA-ES3 to Ago2 protein in HA-VSMCs. The data are expressed as mean ± SD, n=3, *p<0.05, **p<0.005, ***p<0.0005. NG: normal glucose; OC: osmolarity control; HG: high glucose; NC: negative control.

Bioinformatics analysis showed that some complementary sites existed between miR-34c-5p and lncRNA-ES3 (Figure 3A). Moreover, qRT-PCR verified that the level of lncRNA-ES3 was markedly increased in HA-VSMCs treated with HG (Figure 3B). However, OC did not influence the expression of lncRNA-ES3. Hence, we intended to further explore whether miR-34c-5p could directly interact with lncRNA-ES3. Firstly, overexpression of miR-34c-5p by transfecting miR-34c-5p mimics to HA-VSMCs significantly reduced lncRNA-ES3 expression (Figure 3C). When introducing shlncRNA-ES3 to knockdown lncRNA-ES3 expression, we found that the shlncRNA-ES3-2 variant was the most effective to inhibit the expression of lncRNA-ES3 (Figure 3D), hence we chose it for the downstream study. In the knockdown study, the expression of lncRNA-ES3 elevated the expression of miR-34c-5p (Figure 3E). Subsequently, the luciferase reporter assay showed that the overexpression of miR-34c-5p significantly decreased the relative luciferase activity of the WT-lncRNA-ES3 reporter, but miR-34c-5p had no effect on luciferase activity of Mut-lncRNA-ES3 reporter, in which the putative binding sites between lncRNA-ES3 and miR-34c-5p were mutant (Figure 3F). Lastly, biotin-labeled miR-34c-5p pull-down assay was performed to verify that the sequence of miR-34c-5p could highly bind with lncRNA-ES3 (Figure 3G). Additionally, RIP assay was performed using Ago2 antibody to explore whether miR-34c-5p and lncRNA-ES3 were involved in RNA-induced silencing complex (RISC). The results showed that miR-34c-5p and lncRNA-ES3 were substantially enriched by Ago2 antibody compared with control IgG antibody (Figure 3H), which suggested that miR-34c-5p and lncRNA-ES3 were present in RISC. Based on these data, we could draw a conclusion that lncRNA-ES3 and miR-34c-5p could combine with each other in HA-VSMCs.

### BMF was the target of miR-34c-5p

**Figure 4:**
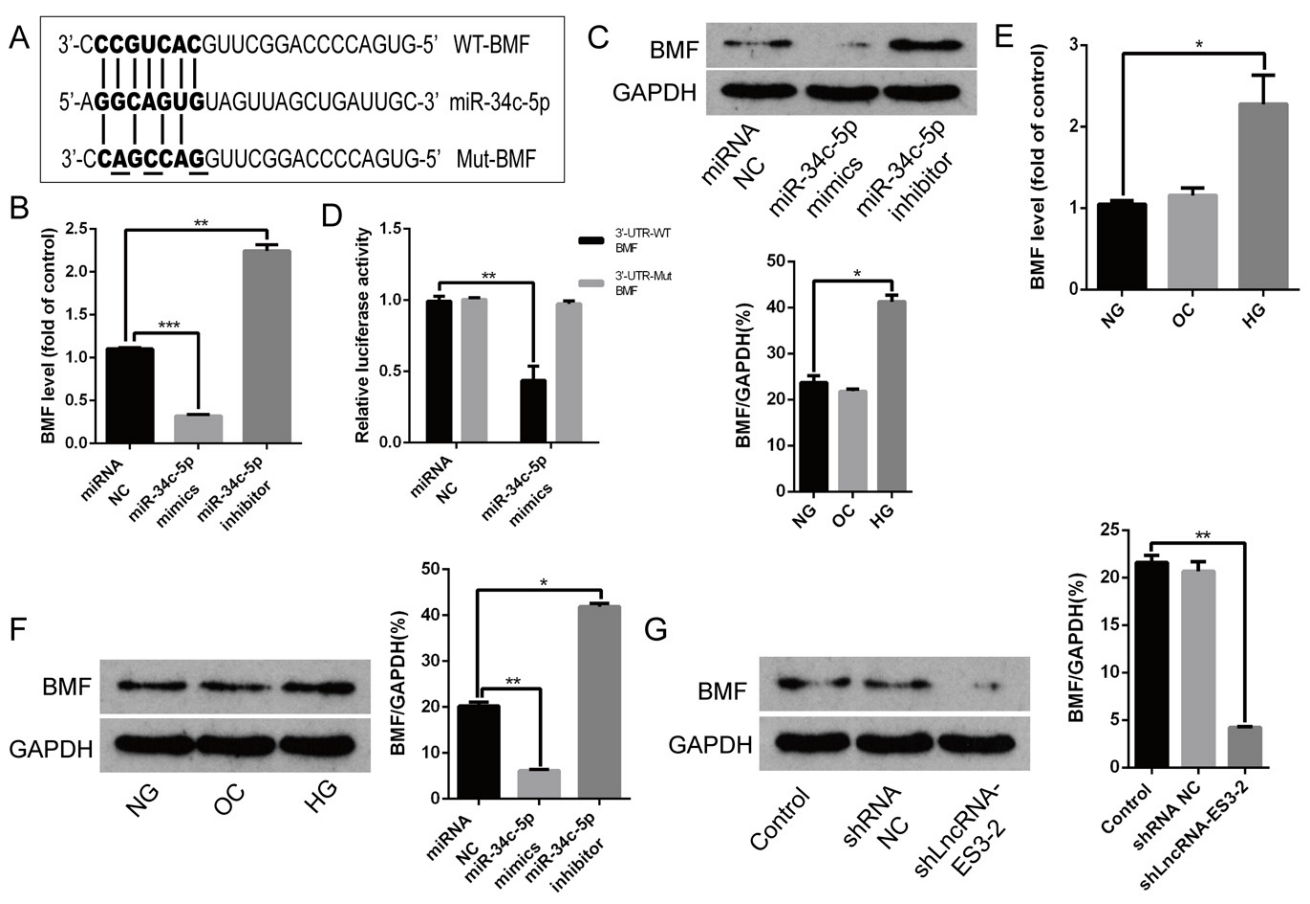
**BMF was the target of miR-34c-5p.** (**A**) Schematic representation of the miR-34c-5p putative target sites in BMF 3’UTR and alignment of miR-34c-5p with WT and Mut BMF 3’UTR showing pairing. The mutated nucleotides were underlined. (**B** and **C**) HA-VSMCs were transfected with miRNA NC, miR-34c-5p mimics, and miR-34c-5p inhibitors, and harvested for the examination of BMF mRNA and protein. (**D**) The WT-BMF 3’UTR and Mut-BMF 3’UTR were co-transfected with miR-34c-5p mimics or control oligos into HA-VSMCs. Forty-eight hours after transfection, luciferase activities were measured. (**E** and **F**) qRT-PCR and Western blot analysis showed the expression level of BMF in HA-VSMCs cultured with NG, OC, and HG. (**G**) HA-VSMCs were transfected with shRNAs NC and shlncRNA-ES3-2, and the protein level of BMF was detected by Western blot. The data are expressed as mean ± SD, n=3, *p<0.05, **p<0.005, ***p<0.0005. NG: normal glucose; OC: osmolarity control; HG: high glucose; NC: negative control.

Bioinformatics analysis showed that the 3’UTR region of BMF was predicted to contain some potential binding sites for miR-34c-5p (Figure 4A). In cultured HA-VSMCs, overexpression of miR-34c-5p significantly reduced both the mRNA and protein level of BMF, while miR-34c-5p inhibitor dramatically increased them (Figure 4B and 4C). The following luciferase assay demonstrated that enforced expression of miR-34c-5p markedly suppressed the luciferase activity of WT-BMF reporter, while no change was observed in the luciferase activity of Mut-BMF reporter after miR-34c-5p overexpression (Figure 4D). In addition, in cultured HA-VSMCs, qRT-PCR assay revealed that the expression of BMF was markedly increased in HA-VSMCs induced by HG compared with NG (Figure 4E). Western blot demonstrated that HG also induced BMF protein level in HA-VSMCs (Figure 4F). Nevertheless, OC had no effect on both the level of mRNA and protein of BMF. Thus, the expression trend of BMF was consistent with that of lncRNA-ES3 in HA-VSMCs under HG. Western blot further showed that silencing of lncRNA-ES3 prominently inhibited BMF expression (Figure 4G). Collectively, these data indicated that BMF was the target of miR-34c-5p and that lncRAN-ES3 acted as a ceRNA of miR-34c-5p to regulate expression of the target gene, BMF, in HA-VSMCs.

### miR-34c-5p inhibited whereas lncRNA-ES3 and BMF promoted calcification/senescence of HA-VSMCs

**Figure 5:**
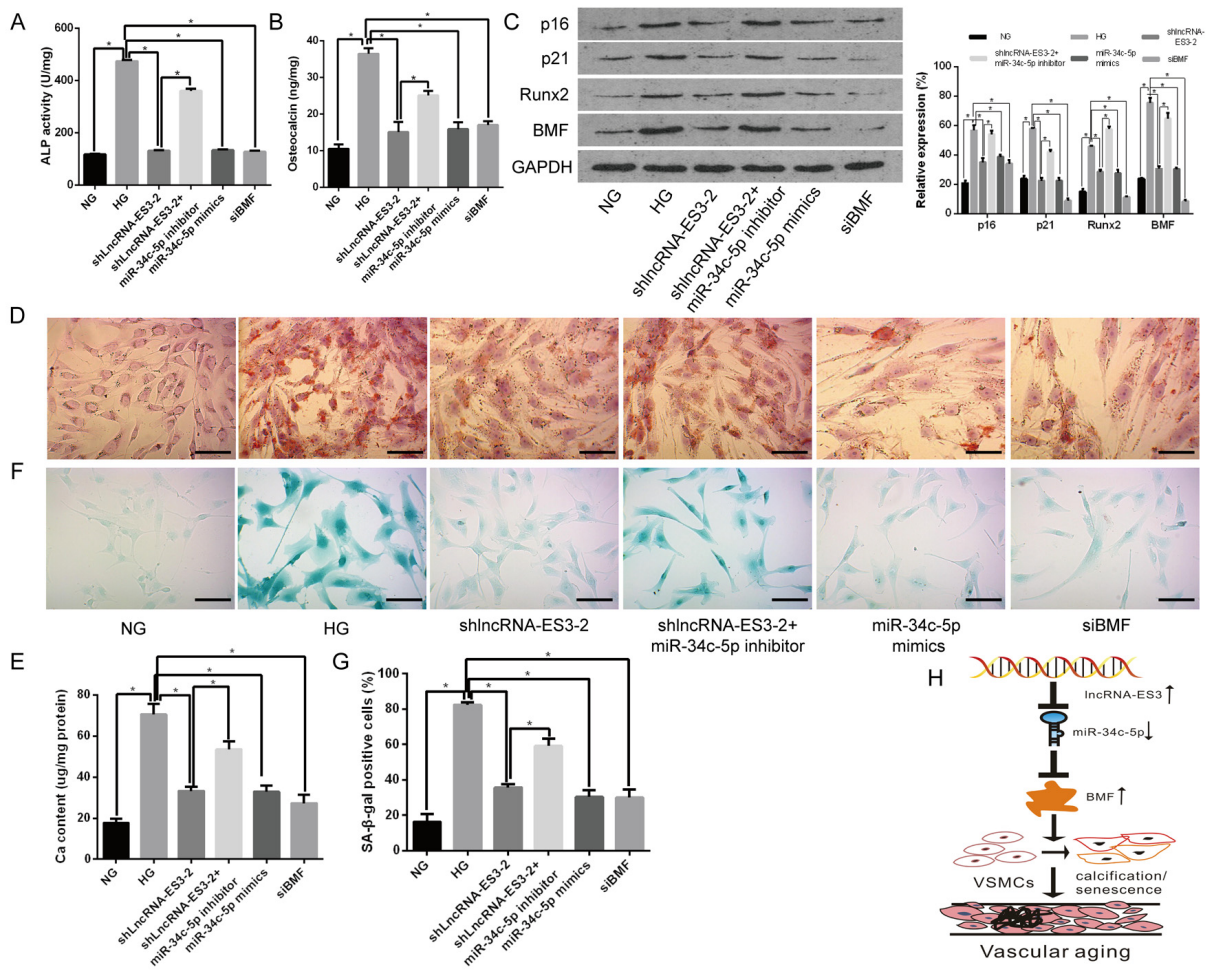
**miR-34c-5p inhibited whereas lncRNA-ES3 and BMF promoted the calcification/senescence of HA-VSMCs.** (**A** and **B**) The ALP activity and OC secretion were detected in HA-VSMCs with different treatment, respectively. (**C**) Representative images of Western blot analyses of p16, p21, Runx2, and BMF in HA-VSMCs with different treatment are shown. (**D** and **E**) Alizarin Red S staining showed the mineralized nodules in HA-VSMCs, and the calcium content was extracted with cetylpyridinium chloride and quantified by spectrophotometry. Representative pictures are shown and the scale bar is 100 μm. (**F** and **G**) SA-β-gal staining showed the senescent cells of HA-VSMCs with different treatment, and the quantification of SA-β-gal-stained positive cells is shown. Representative pictures are shown and the scale bar is 100 μm. (**H**) The model proposed to explain the mechanism of miR-34c-5p in inhibiting VSMC calcification/senescence is shown. Herein, lncRNA-ES3 inhibits miR-34c-5p expression, enhances the expression of BMF, and finally promotes calcification/senescence of VSMCs. The data are expressed as mean ± SD, n=3, *p<0.05. NG: normal glucose; HG: high glucose; NC: negative control.

The biological roles of miR-34c-5p in calcification/senescence of HA-VSMCs were explored. Herein, overexpression of miR-34c-5p attenuated calcification of HA-VSMCs induced by HG, which were verified by a significant decrease of ALP activity, OC secretion, Runx2 expression, and the formation of mineralized nodules (Figure 5A–5E). Meanwhile, the decreased expression of p16 and p21, as well as the staining of SA-β-gal positive cells, confirmed that the senescence of HA-VSMCs transfected with miR-34c-5p mimics was also significantly alleviated (Figure 5C, 5F–5G). To evaluate the effect of lncRNA-ES3 and BMF on the calcification/senescence of HA-VSMCs, the expression of lncRNA-ES3 and BMF in HA-VSMCs were knocked down using shlncRNA-ES3 and siBMF, respectively. The results demonstrated that silencing of lncRNA-ES3 or BMF markedly attenuated the high glucose-stimulated HA-VSMCs calcification/senescence, which were confirmed by the decrease in ALP activity, OC secretion, Runx2, p16, and p21 expression, and mineralized nodules, as well as staining of SA-β-gal positive cells (Figure 5A–5G). Interestingly, the inhibitory effect of lncRNA-ES3 deficiency on HA-VSMCs calcification/senescence was greatly abrogated by miR-34c-5p inhibitor, as presented by the increase in ALP activity, OC secretion, Runx2, p16, and p21 expression, and mineralized nodules, as well as staining of SA-β-gal positive cells (Figure 5A–5G). Taken together, these results indicated that miR-34c-5p downregulation contributed to the calcification/senescence of HA-VSMCs, and that the mechanism might be mediated by lncRNA-ES3 and BMF in high glucose-induced HA-VSMCs.

## DISCUSSION

In the present study, we demonstrated that HG could induced severe calcification/senescence of HA-VSMCs and identified miR-34c-5p could inhibit the calcification/senescence of HA-VSMCs. Mechanistically, we found for the first time that lncRNA-ES3 directly sponged to miR-34c-5p and acted as a ceRNA of miR-34c-5p to enhance the expression of BMF, a target gene of miR-34c-5p (Figure 5G). This finding provides insight into noncoding RNAs in VSMCs calcification/senescence of diabetes and suggests that modulation of the activity of noncoding RNAs such as miR-34c-5p and lncRNA-ES3 may be a novel therapeutic approach to treat human cardiovascular diseases related to diabetes.

Vascular calcification is a salient feature of arterial aging [1, 23]. The phenotypic changes that characterize the aging process are governed by specific alterations in the pools of expressed proteins, such as p21 and p16. Moreover, SA-β-gal staining is a recognized method to determine senescence [24, 25]. Meanwhile, vascular calcification is an important phenotype of vascular aging and is one of the common effects of macrovascular complications in patients with diabetes, mainly involving the media of artery [26, 27]. According to its character of media calcification, VSMCs are considered as the major osteoblast-like cells after undergoing a phenotypic switch, which is manifested by an increase expression of ALP, OC, and Runx2 as well as mineral nodules formation [12, 28]. Our previous studies had done a series of researches for the mechanism of osteoblastic differentiation of VSMCs [2, 27–29]. Increasing number of VSMCs senescence is one of the main reasons leading to vascular aging. Several researches had suggested that vascular calcification could be enhanced by hyperglycemia [30–32] and in the present study, we also found that HG could induce HA-VSMC calcification, which was confirmed by mineralized nodules formation. However, until now, no research had reported to study the role of HG in VSMCs senescence. In our study, interestingly, the SA-β-gal staining as well as increased senescence-associated proteins including p21 and p16 verified that HA-VSMCs cultured in HG occurred in serious senescence. Collectively, our data demonstrated that HG could induce calcification/senescence of VSMCs.

Recently, increasing evidence had shown that numerous miRNAs played important roles in the regulation of proliferation, differentiation, apoptosis, and calcification of VSMCs [7, 12, 33]. MiR-34c, a muscle-specific miRNA, had been considered to be expressed in skeletal muscles and cardiac myocytes, and might play important roles in skeletal and cardiac muscle development, physiology, and disease pathogenesis [9, 10, 34]. Data from Bae showed that miR-34c played a critical role in bone homeostasis in part by modulating notch signaling both *in vitro* and *in vivo* [34]. Moreover, Wei et al. found that miR-34b/c inhibited osteoblast proliferation and differentiation in the mouse by targeting Satb2 [9]. Vast evidence had shown that arterial calcification was an active, complex, and cell-regulated process, which was companied with the phenotypic conversion of VSMCs into osteoblast-like cells [27, 35]. Thus, miR-34c might have an effect on the differentiation of VSMCs. Hao et al. had demonstrated that miR-34b/c can inhibit testosterone-induced VSMCs calcification [10]. However, the specific mechanisms of miR-34c to regulate arterial calcification are still being explored, and whether miR-34c also playing a key role in senescence of VSMCs is still unknown. In the present study, we also found that miR-34c-5p, not miR-34c-3p, was downregulated in HA-VSMCs induced by HG. Furthermore, overexpression of miR-34c-5p decreased ALP activity, OC secretion, Runx2 expression, and mineralized nodule formations. Moreover, the expression of p16 and p21 were decreased significantly and staining of SA-β-gal positive cells was attenuated when overexpressing miR-34c-5p. These results indicated that the process of calcification/senescence of HA-VSMCs was inhibited by miR-34c-5p.

LncRNAs, a class of noncoding RNAs longer than 200 nt in length, was proved to affect cellular senescence broadly [16]. In addition, there is increasing evidence that lncRNAs may play a role in VSMCs functions [36, 37]. For example, lncRNA growth block specificity 5 (GAS5) had been identified as a novel modulator of SMC differentiation by affecting Smad3 function [37], whereas lncRNA-MALAT1 promoted the transformation of smooth muscle cells from contraction to synthetic phenotypes by regulating autophagy [36]. In addition, ceRNAs are stable lncRNAs that accumulate in large numbers and modulate gene expression in different ways, including decoys or sponges for microRNAs [21, 22]. For instance, Lv et al demonstrated that lncRNA H19/miR-675/PTEN was the signaling axis in SMCs proliferation [19]. Another study showed that H19 facilitated proliferation and inhibited apoptosis by sponging to miR-148b in ox-LDL-stimulated HA-VSMCs [20]. Based on these data, we found that some complementary sites existed between miR-34c-5p and lncRNA-ES3. Although some lncRNAs had been reported to regulate a variety of VSMCs functions, no reports had described the role of lncRNA-ES3 on the calcification/senescence of VSMCs before. In our present study, we found that the expression of lncRNA-ES3 was significantly increased in HA-VSMCs treated with HG, while knocking down lncRNA-ES3 resulted in a dramatic suppression of calcification as well as senescence in high glucose-stimulated HA-VSMCs, which suggested that lncRNA-ES3 was involved in regulating calcification/senescence of HA-VSMCs. Moreover, there are three key clues to support the hypothesis that miR-34c-5p directly reacts with lncRNA-ES3 in HA-VSMCs. Firstly, overexpression of miR-34c-5p significantly reduced the expression of lncRNA-ES3; on the other hand, knocking down the expression of lncRNA-ES3 elevated the expression of miR-34c-5p. Secondly, overexpression of miR-34c-5p significantly decreased the relative luciferase activity of the WT-lncRNA-ES3 reporter rather than that of Mut-lncRNA-ES3 reporter. Thirdly, biotin-labeled miR-34c-5p pull-down assay verified that the sequence of miR-34c-5p could be highly bound to lncRNA-ES3. Lastly, the RIP assay showed that miR-34c-5p and lncRNA-ES3 were present in RISC. Nevertheless, the effects of lncRNA-ES3 on calcification/senescence, whether mediated by miR-34c-5p *in vivo* or not, are still needed to be further explored.

Emerging evidence showed that miRNAs often exerted functions by regulating translation or stability of target mRNAs. Previously, BMF was verified to be a target of miR-34c-5p and contributed to resistance to apoptosis induced by paclitaxel in lung cancer [38]. In addition, miR-34c could increase the expression of Bcl-2 and inhibit the apoptosis of renal podocytes in a high glucose environment [13]. Garnet et al. also confirmed that BMF could promote apoptosis of proximal renal tubular cells in mice with diabetic nephropathy [14]. In our present study, we found that the expression of BMF was increased significantly in the process of calcification/senescence of HA-VSMCs induced by HG, and that it was a potential target of miR-34c-5p, which was validated by the luciferase reporter assay. Silencing the expression of BMF could attenuate the calcification and senescence of HA-VSMCs. Moreover, our study demonstrated that lncRNA-ES3 acted as a ceRNA of miR-34c-5p to enhance target gene BMF expression in HA-VSMCs. Taken together, BMF was the target of miR-34c-5p and involved in regulating the calcification/senescence of HA-VSMCs. However, the role of BMF in regulating calcification/senescence of HA-VSMCs, whether dependent on miR-34c-5p or not, is still needed to further illuminate.

In summary, our present study elucidated for the first time that lncRNA-ES3/miR-34c-5p/BMF is the regulatory axis in high glucose-induced HA-VSMCs calcification/senescence. As shown in the scheme in Figure 5H, this links together long noncoding RNAs, microRNAs, and protein encoding genes. The findings not only revealed a novel function of miR-34c-5p and lncRNA-ES3, but also have important diagnostic and therapeutic implications in the setting of calcification/senescence in patients with diabetes. Nevertheless, in-depth studies on the function and mechanisms of miR-34c-5p in the context of VSMCs and vascular calcification/senescence are still imperative to be performed using animal models in the future.

## MATERIALS AND METHODS

### Cell culture

HA-VSMCs were purchased from ATCC (ATCC-CRL-1999). Cells were cultured in Dulbecco’s Modified Eagle’s Medium (DMEM) supplemented with 10% fetal bovine serum (FBS), penicillin (100 U/mL), and streptomycin (100 μg/mL) at 37°C in a humidified atmosphere of 5% CO_2_. HA-VSMCs were incubated at various times with DMEM plus 10% FBS in the presence of 5 mM glucose or 30 mM glucose. In some experiments, 30 mM mannitol was used as OC. The medium was refreshed every 2 days and cells were passaged every 3–4 days.

### Alizarin Red S staining

Alizarin Red S staining was done as previously described [2, 23]. Briefly, HA-VSMCs cultured with 5 or 30 mM glucose or 30 mM mannitol for 14 days were fixed in 4% paraformaldehyde for 30 minutes at room temperature and then stained with 1% (pH 4.2) Alizarin Red S for 1–2 minutes at 37°C. The stained matrix was assessed and photographed using a digital microscope.

### SA-β-gal staining

SA-β-gal staining was performed using a Senescence-associated β-Galactosidase Staining kit (Beyotime Institute of Biotechnology, Shanghai, China) following the manufacturer’s protocol. Briefly, HA-VSMCs cultured with 5 or 30 mM glucose or 30 mM mannitol for 72 hours were fixed in β-galactosidase fixation solution (2% formaldehyde/0.2% glutaraldehyde in PBS) for 5 min and then washed three times with PBS. The cells were stained in SA-β-gal staining solution (pH 6.0) overnight at 37°C. The intensity of positive SA-β-gal staining was determined as previously described [25].

### Measurement of ALP activity and osteocalcin

The confluent HA-VSMCs were washed with PBS twice then the cell layers were scraped into a solution. The cell lysates were homogenized and measured for ALP activity by ALP assay kit (A059-2, Jiancheng, Nanjing) and for osteocalcin secretory by osteocalcin assay kit (H152, Jiancheng, Nanjing) according to the manufacturer’s instructions. ALP activity and osteocalcin were normalized to total cellular protein of the cell layers by the Bradford protein assay as previously described [39].

### Gene expression determined using qRT-PCR

Total RNA was extracted from cultured HA-VSMCs using Trizol Reagent (Invitrogen, 15596-026) [12]. For BMF mRNA and lncRNA-ES3 detection, cDNA was synthesized from 1 μg of total RNA using RevertAid™ H Minus First Strand cDNA Synthesis Kit (Fermentas, K1631). Then, a 20-μl reverse-transcription reaction was carried out for 60 minutes at 42°C, followed by a second step of 10 minutes at 70°C and a final hold at 4°C. Quantitative PCR analysis was performed with SYBR Green PCR Master Mix (ABI 4309155) in a real-time fluorescence quantitative PCR instrument (ABI, 7900, USA). For qPCR analysis, 25-μl reactions were incubated in a 96-well optical plate at 95°C for 5 minutes, followed by 40 cycles of 95°C for 20 seconds, 60°C for 20 seconds, and 72°C for 20 seconds. Data were normalized to GAPDH values.

For miR-34c analysis, total RNA was purified by miRNeasy Mini Kit (Qiagen, 217004), and miRNA Q-PCR Detection Kit (Genecopoiea, R0101L) was used as described by the manufacturer’s protocol using U6 snRNA as the reference. Briefly, a 25-μl reverse-transcription reaction was carried out for 60 minutes at 37°C, 5 minutes at 85°C, and a hold at 4°C. qPCR was performed for 5 minutes at 95°C, followed by 40 cycles of 10 seconds at 95°C, 20 seconds at 60°C, and 10 seconds at 72°C. All of the PCR primers used in this study are shown in Supplementary Table 1. The relative standard curve method (2^-△△CT^) was used to determine the relative mRNA and miRNA expression. Results were expressed as fold change relative to the appropriate control. The qPCRs were run in triplicate and results are presented as the mean ± standard error of samples.

### RNA interference

MiR-34c-5p mimics, inhibitor, and their negative controls were purchased from RiboBio (Guangzhou, China). ShlncRNA-ES3 (shlncRNA-ES3 #1,2,3,4), si-BMF, and their negative controls (si-con) were designed and synthesized by GenePharma Co. Ltd (Shanghai, China). The sequences of shlncRNA-ES3 and siBMF used in this study are shown in Supplementary Table 2. The cells were transfected using Lipofectamine 3000 (Invitrogen, USA). Lastly, the inhibitory efficiency of siRNAs was verified by qRT-PCR and the most effective shlncRNA-ES3 was used for the downstream functional experiments.

### Western blot analysis

Protein expression analysis was detected by Western blot analysis as previously described [29, 40]. The membranes were incubated with primary antibodies, including anti-BMF (ab9655, 1:1000, Abcam), anti-Runx2 (ab76956, 1:1000, Abcam), anti-p16 (10883-1-AP, 1:1000, Proteintech), anti-p21 (10355-1-AP, 1:1000, Proteintech), and anti-GAPDH (sc-365062, 1:3000, Santa Cruz Biotechnology) at 4°C overnight, followed by incubation with the horseradish peroxidase-conjugated goat anti-rabbit (sc-2004, 1:5000, Santa Cruz Biotechnology) or goat anti-mouse (sc-2005, 1:5000, Santa Cruz Biotechnology) secondary antibodies at 37°C for 1 hour. The immunoreactive bands were visualized using the ECL Plus Western blot detection kit (Amersham Biosciences U.K. Ltd) and densitometric quantification of band intensity from three independent experiments was carried out with the Image-Pro Plus 6.0 software. The relative expression level of target protein was normalized to the intensity of the GAPDH band.

### Luciferase reporter assay

Luciferase reporter assay was performed to determine the binding of lncRNA-ES3 and miR-34c-5p as well as miR-34c-5p and BMF. Partial fragments of lncRNA-ES3 and BFM 3’UTR containing the predicted binding sites of miR-34c-5p were amplified by PCR and cloned into XbaI-FseI restriction sites of the pGL3 luciferase reporter vector (Promega, Madison, WI, USA). Meanwhile, the QuikChange Multi Site-Directed Mutagenesis kit (Stratagene, Lajolla, CA, USA) was employed to construct a mutant 3’UTR of lncRNA-ES3 and BMF. HA-VSMCs were co-transfected with a luciferase reporter carrying wild type lncRNA-ES3 3’UTR (WT-pGL3-lncRNA-ES3), mutant lncRNA-ES3 3’UTR (Mut-pGL3-lncRNA-ES3), wild type BMF 3’UTR (WT-pGL3-BMF), mutant BMF 3’UTR (Mut-pGL3-BMF), and miR-34c-5p mimics or scrambled oligos, respectively. Then, 48 hours after transfection, luciferase activities in cells were quantified via a luciferase reporter assay system (Promega) according to the protocols of the manufacturer.

### RNA pull-down assay

RNA pull-down assays were performed using Pierce™ Magnetic RNA-Protein Pull-Down Kit (Thermo Fisher Scientific, 2016420). Briefly, biotin-labeled RNAs were transfected into HA-VSMCs and the cells were lysed using Thermo Scientific Pierce IP lysis buffer. Biotinylated RNAs were mixed and incubated with HA-VSMCs cell lysates. Streptavidin magnetic beads were added to each binding reaction and the magnetic beads were washed twice with 0.1 M NaOH and 50 mM NaCl. The magnetic beads were then resuspended in equal volume of 20 mM Tris (pH 7.5) and incubated with RNA-protein binding reaction buffer for 60 minutes at 4°C. Then, the bound RNA-protein complexes were washed and eluted from the magnetic beads. Lastly, RNAs in the complexes were purified and qRT-PCR assay was employed to measure the enrichment patterns of lncRNA-ES3 and miR-34c-5p.

### RNA immunoprecipitation (RIP) assay

RIP assay were performed using EZ-Magna RIP kit (No. 17-701, Millipore, Billerica, MA, USA) and Argonaute 2 (Ago2) antibody (Abcam, ab32381) to explore whether miR-34c-5p and lncRNA-ES3 existed in RNA-induced silencing complex (RISC). Anti-SNRNP70 was taken as the positive control. Briefly, HA-VSMCs cells were lysed in RIP lysis buffer, followed by the incubation of protein A/G magnetic beads and antibody against rabbit IgG or Ago2. Then, RNAs in magnetic beads-binding complexes were purified. Lastly, qRT-PCR assay was employed to measure the enrichment patterns of miR-34c-5p and lncRNA-ES3 by IgG or Ago2 antibody.

### Statistical analysis

The data are presented as mean ± standard deviation (SD) and were analyzed using GraphPad Prism software (GraphPad Prism version 6.0). The normality of data distribution was assessed before analysis. Student’s t-test was used to compare normally distributed data between two different groups, while one-way analysis of variance (ANOVA) was used for multiple groups. A level of *p<*0.05 was considered statistically significant. All experiments were repeated at least three times, and representative experimental results are shown in the figures.

## SUPPLEMENTARY MATERIALS

Supplementary Tables
