# Supplementary material for: lncRNA-ES3/miR-34c-5p/BMF axis is involved in regulating high-glucose-induced calcification/senescence of VSMCs
Source: Aging (Albany NY). 2019 Jan 17;11(2):523–35. doi: 10.18632/aging.101758 (PMC6366973; doi:10.18632/aging.101758)
Supplement: Supplementary Tables [file aging-11-101758-s001.pdf]

SUPPLEMENTARY MATERIAL

Supplementary Table 1. Nucleotide sequences of primers used in the research

Gene	Primer sequence (5’ to 3’)
BMF	Forward: CCACCAGCCAGGAAGACAAAG<br>Reverse: TGCTCCCCAATGGGCAAGACT
LncRNA-ES3	Forward: ACAAAGCCTGTTTGGTGGTC<br>Reverse: GCTGCCAGATGAGTTGAAC
GAPDH	Forward: GGAGCGAGATCCCTCCAAAAT<br>Reverse: GGCTGTTGTCATACTTCTCATGG
miR-34c-5	Forward: ACACTCCAGCTGGGAGGCAGTGTAGTTAGCT<br>Reverse: CTAACTGGTGTCGTGGAGTCGGCAATTCAGTTG<br>AGGCAATCAG
miR-34c-3p	Forward: ACACTCCAGCTGGGATCACTAACCACACG<br>Reverse: CTCAACTGGTGTCGTGGAGTCGGCAATTCAGTT<br>GAGCCTGGCCG
U6snRNA	Forward: CTCGCTTCGGCAGCACA<br>Reverse: AACGCTTCACGAATTTGCGT

Supplementary Table 2. The sequence of siRNA used in the research

Name	Sequence
shlncRNA-ES3-1	Sense:<br>5’-CACCGGTATCCTCTTAGACTCTCAATTCAAGAGATTGAGAGTCTAAGAGGATACCTTTTTTG -3’<br>Antisense:<br>5’-GATCCAAAAAAGGTATCCTCTTAGACTCTCAATCTCTTGAATTGAGAGTCTAAGAGGATACC-3’
shlncRNA-ES3-2	Sense:<br>5’-CACCGGTTTGGATGACAAATTCTGTTTCAAGAGAACAGAATTTGTCATCCAAACCTTTTTTG -3’<br>Antisense:<br>5’-GATCCAAAAAAGGTTTGGATGACAAATTCTGTTCTCTTGAAACAGAATTTGTCATCCAAACC -3’
shlncRNA-ES3-3	Sense:<br>5’-CACCGGTTAGTTTCCACTAGAAAGTTTCAAGAGAACTTTCTAGTGGAAACTAACCTTTTTTG-3’<br>Antisense:<br>5’-GATCCAAAAAAGGTTAGTTTCCACTAGAAAGTTCTCTTGAAACTTTCTAGTGGAAACTAACC-3’
shlncRNA-ES3-4	Sense:<br>5’-CACCGGTTAGTTTCCACTAGAAAGTTTCAAGAGAACTTTCTAGTGGAAACTAACCTTTTTTG-3’<br>Antisense:<br>5’-GATCCAAAAAAGGTTAGTTTCCACTAGAAAGTTCTCTTGAAACTTTCTAGTGGAAACTAACC-3’
siBMF	Sense:<br>5’-AAGGUGUCAUGCUGCCUUGUTT-3’<br>Antisense:<br>5’-ACAAGGCAGCAUGACACCUUTT-3’
